# The Chronic Effect of Interval Training on Energy Intake: A Systematic Review and Meta-Analysis

**DOI:** 10.1155/2018/6903208

**Published:** 2018-04-01

**Authors:** Jenna Taylor, Shelley E. Keating, David J. Holland, Jeff S. Coombes, Michael D. Leveritt

**Affiliations:** School of Human Movement and Nutrition Sciences, The University of Queensland, St. Lucia, Brisbane, QLD 4072, Australia

## Abstract

Single bouts of acute exercise do not appear to increase subsequent energy intake (EI), even when energy deficit is large. However, studies have shown a compensatory effect on EI following chronic exercise, and it remains unclear whether this is affected by exercise intensity. We investigated the chronic effect of high-intensity interval training (HIIT) and sprint interval training (SIT) on EI when compared with moderate-intensity continuous training (MICT) or no exercise (CON). Databases were searched until 13 March 2017 for studies measuring EI in response to chronic exercise (≥4 weeks of duration) of a high-intensity interval nature. Meta-analysis was conducted for between-group comparisons on EI (kilojoules) and bodyweight (kg). Results showed large heterogeneity, and therefore, metaregression analyses were conducted. There were no significant differences in EI between HIIT/SIT versus MICT (*P*=0.282), HIIT/SIT versus CON (*P*=0.398), or MICT versus CON (*P*=0.329). Although bodyweight was significantly reduced after HIIT/SIT versus CON but not HIIT/SIT versus MICT (in studies measuring EI), this was not clinically meaningful (<2% mean difference). In conclusion, there is no compensatory increase in EI following a period of HIIT/SIT compared to MICT or no exercise. However, this review highlights important methodological considerations for future studies.

## 1. Introduction

Obesity is a now a global epidemic with the prevalence of overweight and obesity estimated as 39% of the world's population [1]. Increasing energy expenditure through exercise to manage obesity is a logical approach. However, when compared to dietary restriction, exercise is often portrayed as a somewhat “futile method for weight loss” because exercise interventions commonly lead to less than expected weight loss [2, 3]. Indeed, it is generally accepted that exercise requires concomitant dietary modification for significant weight loss (>5% reduction) [4, 5].

The modest effect of exercise interventions on weight loss is likely a result of individual variability in response to intervention [3]. Proposed mechanisms for less than expected weight loss in some individuals may be explained by reduced exercise adherence [4], decreased spontaneous physical activity [2], overestimation of weight loss from predictive equations [6], and automatic metabolic adaptations that lower the energy expenditure [2]. A comprehensive review of these factors has been presented elsewhere [2]. Dietary compensation factors that could increase caloric intake and result in less than expected weight loss with exercise include biological changes driving appetite [7], changes in food preferences and food reward [8], and other psychological dietary behaviours (e.g., restraint and disinhibition) [9, 10].

There is now a general consensus that acute moderate-intensity aerobic exercise does not increase absolute energy intake [11, 12], even when energy deficit is large [13]. However, there is still uncertainty about the effect of mode and intensity of acute exercise on appetite and energy intake response. There is also less agreement about the effect of higher-intensity exercise and chronic exercise on energy intake. Donnelly and colleagues found that long-term exercise did not result in a compensatory increase in energy intake [12], while a partial compensation (+30%) in energy intake with chronic exercise has been demonstrated following 7 days [14] and 14 days [15].

There is much interest in the effect of high-intensity interval training (HIIT) or sprint interval training (SIT) on weight loss and specifically whether exercise intensity influences appetite and energy intake. Regarding fat loss, results to date are conflicting with studies showing a superior effect of HIIT [16–18], superior effect of MICT [19, 20], and similar effects [21–26]. A recent meta-analysis found no significant differences between HIIT/SIT and MICT on total fat mass or total body fat percentage [27] and that body fat reduction tended to favor MICT if it produced a greater energy expenditure.

The evidence regarding the effect of exercise intensity on appetite and energy intake is also conflicting [26, 28–31]. Appetite is a physical desire for food, mediated by central and peripheral systems (including secretion of hormones via the gastrointestinal tract) that influence both psychological (desire and enjoyment) and physiological (hunger and satiety) states that affect energy intake. In terms of acute studies, only one study [28] has found a significant reduction in ad libitum energy intake following HIIT compared with MICT, despite no differences in perceived appetite. Other studies have found no group differences in energy intake with [30, 32] or without [29, 33] changes in appetite. Following chronic exercise, Sim et al. [26] found a clinically meaningful improvement in appetite regulation following 12 weeks of HIIT but not for MICT and CON. This study measured appetite regulation as ad libitum energy intake following a preload meal. Alkahtani et al. [31] also found an effect of exercise intensity on dietary compensation following a 4-week intervention, with HIIT decreasing intake of fat by 16% and decreasing exercise-induced preference for high-fat, nonsweet foods. The moderate-intensity group increased fat intake by 38% and showed an increase in exercise-induced preference for high-fat, nonsweet foods. Panissa et al. [24] found no group differences in energy intake or appetite after 6 weeks of training.

Acute suppression of appetite from exercise may be due to changes in appetite-regulating hormones, by simultaneously suppressing acylated ghrelin (orexigenic hormone) and increasing peptide YY (anorexigenic hormone) up to 9 hours after exercise [34]. However, despite appetite being suppressed in the hours following exercise, total daily energy intake appears to be unaffected [35]. Therefore, acute appetite suppression may not necessarily translate into decreased volitional caloric intake [13, 36, 37]. Long-term adaptations that may influence appetite and energy intake include changes in psychological eating behaviours (restraint, desire, and food preferences) and improvements in insulin sensitivity. Insulin levels have been shown to improve satiety and reduce energy intake; however, this effect is blunted in people with insulin resistance [38]. Therefore, exercise protocols producing enhanced insulin sensitivity such as HIIT compared with MICT [39] may offer greater improvements in appetite regulation and energy intake [26].

Given the importance of obesity management and the interest in HIIT, we sought to determine what effect this time-efficient exercise option may have on energy intake. HIIT/SIT is often considered time efficient, when greater or comparable health and fitness benefits to MICT are produced with less time commitment [19].

The purpose of this study was to conduct a systematic review with a meta-analysis of randomized controlled trials to determine the chronic effect of HIIT/SIT on energy intake when compared with MICT or no exercise (CON) in an adult population. Understanding the impact of exercise intensity on energy intake would assist in developing more targeted and effective exercise protocols for weight management in the future.

## 2. Methods

This systematic review has been designed and presented in accordance with the Preferred Reporting Items for Systematic Reviews and Meta-Analyses (PRISMA) Statement [40].

### 2.1. Eligibility Criteria

Full-text, randomized controlled trials in English peer-reviewed journals were eligible for inclusion in this systematic review and meta-analysis if energy intake was measured in response to chronic exercise training of a high intensity, or sprint interval nature, compared with MICT or no exercise. Specifically, HIIT was characterized by an exercise intensity of 85–95% peak heart rate (HR_peak_), or 80–95% peak oxygen consumption (VO_2peak_) or peak work rate. SIT was characterized by an exercise intensity relating to all-out sprint, or ≥100% VO_2peak_ or peak power. These are consistent with recently proposed classifications for interval training [41]. Eligibility of studies was not restricted by duration of intervals or all-out sprints. In comparison, MICT was characterized as exercise performed continuously at a steady state, within the intensity range of 60–75% HR_peak_, 40–64% VO_2peak_, or 40–59% heart rate reserve (HRR) [42, 43]. Energy intake (measured as kilojoules or calories) was required to be measured before and after the exercise intervention. There was no restriction placed on assessment methods for energy intake. Therefore, if the study reported energy intake, it was included in the meta-analysis. To be considered a chronic exercise intervention, duration was required to be ≥4 weeks. Studies were excluded if participants were children or adolescents or if participants were provided with individual dietary prescription or medical weight loss aids.

### 2.2. Search Strategy

The search strategy involved major databases (PubMed, Scopus, and EMBASE/MEDLINE) and the following search terms in title/abstract/keywords: “exercise” OR “training” AND “interval” OR “intermittent” OR “sprint” OR “low volume” AND “energy intake” OR “caloric intake” OR “body composition” OR “bodyweight”, AND “week/s” OR “month/s”. Additional search criteria were applied to retrieve studies published in English and to eliminate reviews and studies involving animals. The last search was conducted on March 13, 2017.

### 2.3. Data Extraction

Full-text versions were independently assessed for eligibility criteria and quality by 2 investigators (Jenna Taylor and Michael D. Leveritt) and coded as “yes” or “no.” If a discrepancy in coding between authors was presented, a discussion took place until the result was unanimous. An independent researcher was available if a unanimous decision could not be reached. The outcome measures used were energy intake in kilojoules. If data were reported in kilocalories, these were converted to kilojoules using a conversion factor of 4.18 kilojoules per kilocalorie. The secondary outcome measure of bodyweight in kilograms was also extracted from the studies reporting energy intake. Data on participant characteristics and other aspects of study methodology (including exercise interventions and energy intake method) were also extracted into table format.

### 2.4. Study Quality

Quality of the included studies was assessed by a modified Physiotherapy Evidence Database (PEDro) Scale [44]. The original 11-point scale includes items for eligibility criteria, random allocation, concealment of allocation, baseline comparability, blinding of subjects, blinding of therapists, blinding of assessors, adequate follow-up, intention to treat analysis, between-group statistical comparisons, and reporting of point estimates and variability. The scale was modified to include an additional 4 criteria that may have impact on the primary outcome: exercise training supervision, reporting of exercise adherence, measurement of habitual physical activity, and estimation/calculation of energy expenditure. Additionally, the scale was modified to exclude 2 criteria that are technically challenging in interval training exercise interventions: blinding of subjects and blinding of therapists. Therefore, the modified scale included 13 criteria with one point awarded for each.

### 2.5. Meta-Analyses

All analyses were conducted using the Comprehensive Meta-Analysis software (Version 3, Biostat, Englewood, NJ). Methods for meta-analysis and metaregression were based on recommendations for continuous outcomes from the Cochrane Handbook for Systematic Reviews [45].

For the primary analysis, a between-group meta-analysis was conducted by pooling energy intake for HIIT/SIT compared with MICT, HIIT/SIT compared with CON, and MICT compared with CON. The between-trial standardized mean difference or effect size, 95% confidence interval (CI), and *P* values were calculated using a random effect model. We presumed a correlation of 0.7 between outcomes measured within each comparison group. Hedge's g was used to measure the effect size due to small sample sizes of the included studies. Heterogeneity was assessed via the Cochran Q-test (significance determined at the level of significance *P*=0.1) and quantified with the *I*^2^ statistic to determine how much of the variability between studies is due to heterogeneity rather than chance. For the secondary analysis, the same methods and comparisons were used for bodyweight data. Publication bias and small-study effect was assessed by visual inspection of funnel plot asymmetry (precision versus effect size) using Egger's test and by calculating the *P* value of Egger's intercept. Metaregression analyses were performed to examine the relationships between effect size estimates and the following covariates: (1) duration of intervention, (2) the total number of exercise sessions, (3) age, (4) sex, (5) baseline body mass index (BMI), (6) metabolic disease, (7) energy intake collection method, and (8) study quality.

## 3. Results

**Figure 1:**
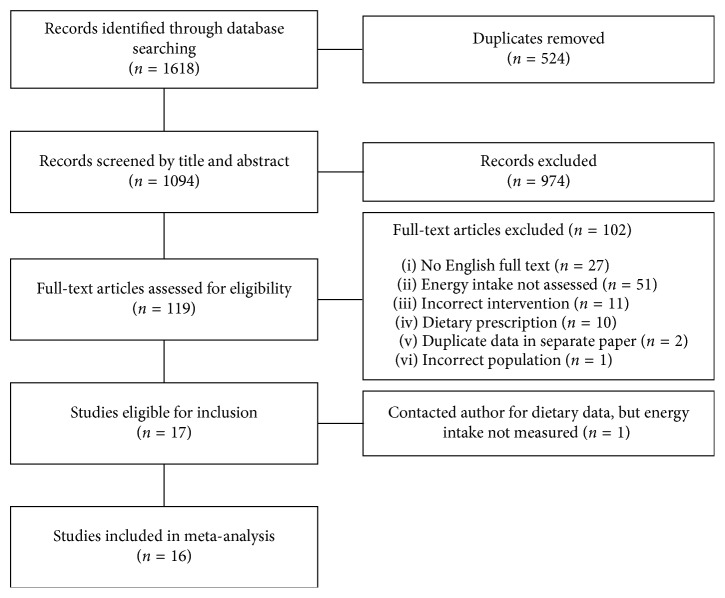
Flowchart of study selection.

Results of the search strategy and study selection are outlined in Figure 1. A total of 1618 studies were retrieved using the aforementioned search strategy with 1094 studies remaining following removal of duplicates. During title and abstract screening, 974 studies were excluded, leaving a total of 119 studies for full-text analysis. Of the 119 studies, English full-text versions were available for 92 studies. After exclusions, 17 studies remained following full-text analysis, with 6 authors being contacted for energy intake data [20, 25, 26, 46–48]. Of the authors contacted, one study had not measured energy intake [25], leaving 16 studies to be included in the meta-analysis. One of the contacted authors reported that their energy intake data were published elsewhere [49], and therefore, this paper was used in the analysis.

### 3.1. Participant Characteristics

Participant characteristics are summarized in Table 1. Data were reported for a total of 475 participants in the included studies (159 HIIT, 64 SIT, 165 MICT, and 87 CON). The mean age of participants ranged from 20 to 55 years. Six studies recruited only males [17, 21, 23, 26, 47, 50], six studies had only females [18, 20, 24, 51–53], and four studies recruited both males and females [19, 46, 49, 54]. Only one study mentioned timing of the menstrual cycle [18], and no studies reported the use of oral contraceptives. Most studies used healthy individuals [18–21, 24, 26, 46, 50–54], with four studies recruiting participants with metabolic syndrome [17, 23, 49] or type 2 diabetes [47]. Only one study recruited active participants [53]. All other studies recruited inactive participants.

### 3.2. Study Methodology Characteristics

Exercise intervention characteristics are detailed in Table 1. Sixteen studies were included in the meta-analysis with a total of 12 HIIT groups, 13 MICT groups, 4 SIT groups, and 7 CON (no exercise) groups. Due to the small number of SIT groups, these were combined with HIIT for analysis, giving a total of 16 HIIT/SIT groups.

Large heterogeneity of protocols existed within the HIIT/SIT groups, with some studies using high-intensity intervals of <30 seconds [18, 26, 46, 47, 50, 52], 30–60 seconds [19, 21, 24, 51, 54], 2 minutes [17, 53], 3 minutes [23], and 4 minutes [20, 49]. On the contrary, the intensity of exercise prescribed for HIIT was consistent at 85–95% VO_2peak_ or HR_peak_. The only exception was the SIT studies that employed workloads of 120% VO_2peak_ [19], 170% VO_2peak_ [26], or “all-out sprinting” [47, 51]. MICT groups were also relatively similar with regard to exercise intensity with prescriptions in the range of 50–70% VO_2peak_ or HR_peak_.

Participants exercised at a frequency of 4 days per week [17, 52, 54] or 3 days per week [18–21, 23, 24, 26, 46, 47, 49–51, 53], with duration of exercise <30 minutes per day [21, 24, 47, 50–53] or 30–50 minutes per day [17–20, 23, 26, 49, 54]. Nine studies [17, 18, 20, 21, 24, 26, 46, 51, 54] reported using various methods to create “isocaloric” or “work-matched” protocols for HIIT and MICT. Most studies reported supervision of training [17, 19, 23, 24, 26, 46–48, 50, 51, 53, 54]; however, adherence to training intensities was poorly reported. Only one study confirmed adherence to the prescribed intensity of training protocols [17]. Three studies reported a significant difference in average training workload between groups but did not discuss whether this adhered with the intended intensity [18, 26, 47, 50]. One study reported no significant difference in average training HR between groups [52], and the remaining 11 studies made no mention of average training intensity or adherence [19–21, 23, 24, 46, 49, 51, 53, 54].

Studies also varied in their measurement of daily energy intake. The majority of studies used self-reported food diaries for 3 days [18, 19, 23, 24, 46, 47, 50, 52, 53] or 7 days [54]. Other studies used a self-administered diet history [21] or food frequency questionnaires [17, 20, 49]. One study used an ad libitum test meal with known composition and then used the self-reported weighed food diary for remainder of the day [26], and one study used the automated 24-hour diet recall method over 3 days [51].

All studies except Earnest et al. [17] included means and standard deviations of daily energy intake before and after the intervention. Earnest et al. [17] reported the mean and standard deviation of baseline energy intake and change scores for postintervention energy intake.

### 3.3. Study Quality

Using the modified PEDro Scale, six studies scored ≤8/13 [18, 20, 21, 24, 50, 52] and ten studies scored ≥8/13 [17, 19, 23, 26, 46, 47, 49, 51, 53, 54]. All included studies randomly allocated participants to groups, reported statistical comparisons between groups for the primary outcome, and reported point estimates and variability. Only one study reported blinding the assessor [19]. Details of study quality scores are presented in Table 2. As the majority of studies scored ≥8, metaregression was performed to assess the study quality; however, the results showed no significance.

### 3.4. Meta-Analyses

**Figure 2:**
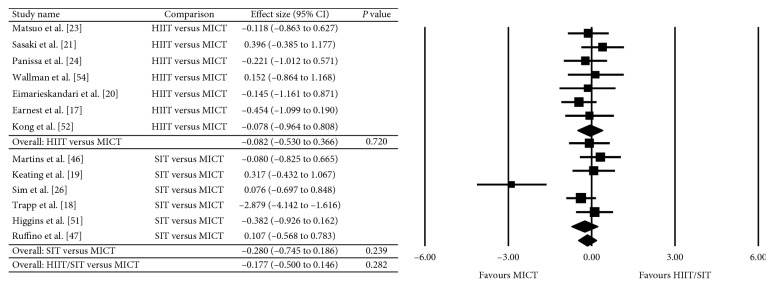
Meta-analysis for the comparison of HIIT/SIT versus MICT on energy intake. HIIT: high-intensity interval training, SIT: sprint interval training, MICT: moderate-intensity continuous training, and CI: confidence interval.

**Figure 3:**
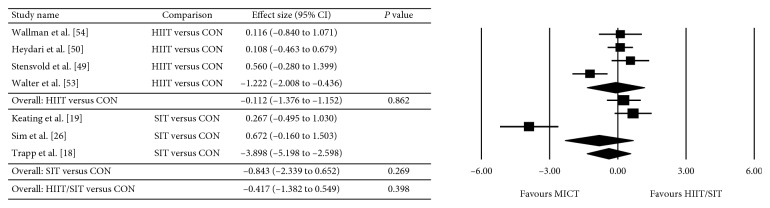
Effect of HIIT/SIT versus CON on energy intake. HIIT: high-intensity interval training, SIT: sprint interval training, CON: control, and CI: confidence interval.

**Figure 4:**
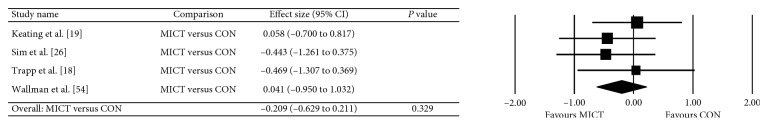
Effect of MICT versus CON on energy intake. MICT: moderate-intensity continuous training, CON: control, and CI: confidence interval.

**Figure 5:**
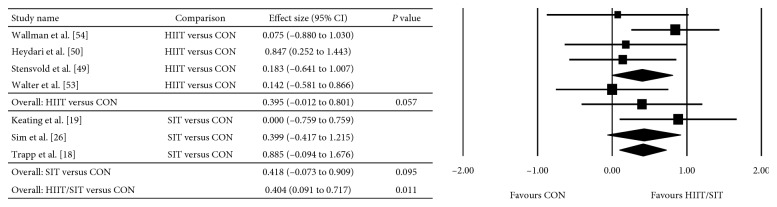
Meta-analysis for the comparison of HIIT/SIT versus CON on bodyweight. HIIT: high-intensity interval training, SIT: sprint interval training, CON: control, and CI: confidence interval.

**Figure 6:**
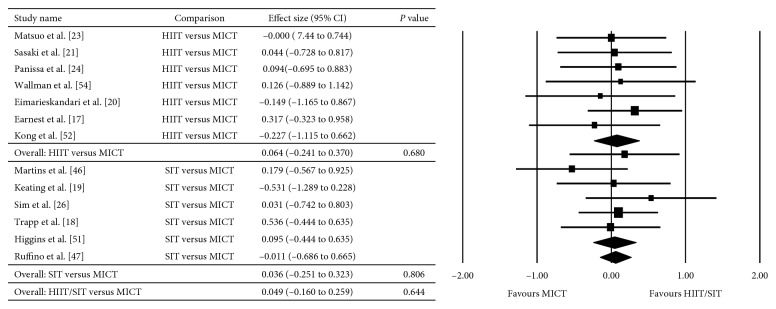
Effect of HIIT/SIT versus MICT on bodyweight. HIIT: high-intensity interval training, SIT: sprint interval training, MICT: moderate-intensity continuous training, and CI: confidence interval.

**Figure 7:**
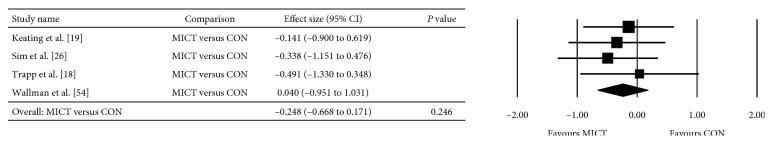
Effect of MICT versus CON on bodyweight. MICT: moderate-intensity continuous training, CON: control, and CI: confidence interval.

The between-group analyses for the primary analysis on energy intake are presented as HIIT/SIT versus MICT (Figure 2), HIIT/SIT versus CON (Figure 3), and MICT versus CON (Figure 4). There were no significant differences in energy intake for any comparison groups, including HIIT/SIT versus MICT (*P*=0.282), HIIT/SIT versus CON (*P*=0.398), or MICT versus CON (*P*=0.329), or with subgroup analyses for HIIT and SIT. There was evidence of moderate heterogeneity for HIIT/SIT versus MICT (*I*^2^ = 50%), high heterogeneity for HIIT/SIT versus CON (*I*^2^ = 92%), and low heterogeneity for MICT versus CON (*I*^2^ = 0%). Due to the heterogeneity of studies, metaregression was performed but found no effect of study duration, the total number of exercise sessions, age, sex, baseline BMI (>30 kg·m^−2^), metabolic disease (compared with healthy participants), energy intake collection method, or study quality (≤8 or ≥8). Outcomes of the metaregression are presented in Table 3. The between-group analyses for the secondary analysis on bodyweight are presented as HIIT/SIT versus CON (Figure 5), HIIT/SIT versus MICT (Figure 6), and MICT versus CON (Figure 7). A significant difference between groups for change in bodyweight was found for HIIT/SIT versus CON (*P*=0.011) but not for HIIT/SIT versus MICT (*P*=0.644) or MICT versus CON (*P*=0.246). There was evidence of low heterogeneity for all comparisons (*I*^2^ = 0%), and therefore, metaregression was not warranted.

Finally, funnel plots and Egger's intercept showed no evidence of publication bias or small-study effect for any primary or secondary analysis comparisons.

## 4. Discussion

To our knowledge, this is the first systematic review with meta-analysis examining the chronic effect of interval training on energy intake when compared with MICT or no exercise. When data from studies were pooled, there were no significant differences in change in energy intake with HIIT/SIT or MICT, and the metaregression found no impact of other variables on these outcomes. Our secondary analysis found a significant reduction in bodyweight with HIIT/SIT compared to CON (in studies that measured energy intake); however, there is no significant difference in bodyweight for HIIT/SIT compared to MICT, or MICT compared to CON.

Our results are consistent with Donnelly et al. [12], who found that 94% of studies showed no effect of long-term continuous aerobic exercise on daily energy intake [12]. On the contrary, Stubbs et al. [14] and Whybrow et al. [15] have demonstrated chronic exercise (>7–14 days) results in partial compensation in energy intake (∼30%) when energy expenditure from exercise is consistent in the realm of 1.5–3.0 MJ/day. Differences in energy intake compensation between our results and these studies are likely explained by the energy cost of exercise. In the included studies, participants exercised 3-4 days per week with exercise duration up to 50 minutes per day. Only 6 studies [17, 18, 23, 26, 46, 54] quantified and reported energy cost of exercise, with all equating to ≤1.5 MJ per session (including basal energy expenditure) and <4.5 MJ per week. This is vastly different from the studies showing a partial compensation in energy intake [14, 15] as participants were exercising 7 days per week for a minimum of 80 minutes/day. Stubbs et al. [14] observed that women partially compensated their energy intake (∼+30%) when the daily energy cost of exercise increased to 3.4 MJ/day over 7 days (23.8 MJ/week). Furthermore, Whybrow et al. [15] demonstrated the same in men when the daily energy cost of exercise increased to 2.8 MJ/day over 14 days (19.6 MJ/week).

Methods of quantifying energy intake in the included studies were variable and subject to potential error and bias, with most studies employing self-reported food diaries or food frequency questionnaires. Self-reported food diaries have an inherent risk of bias as the process of recording food intake can lead participants to change their eating behaviours [55]. Additionally, there is a large participant burden in keeping the record, which may lead to incomplete data. Food frequency questionnaires involve less participant burden but are designed to estimate usual dietary intake of specific food items or nutrients over time (usually 6 to 12 months) rather than daily energy intake [55]. Unfortunately, all methods of assessing energy intake are susceptible to underreporting from participants. However, 24-hour recalls have shown less underreporting (∼12–20%) compared to food frequency questionnaires (∼30–36%) when compared to total energy expenditure by doubly labeled water [56]. Other studies using a multiple-pass 24-hour recall method to measure energy intake have found levels of underreporting to be ∼11% [57]. Using the multiple-pass 24-hour recall method on multiple days has also been shown to reduce underreporting from 30% (1 day) to 11% (2 days) and 4% (3 days) [58]. Inherent variability associated with underreporting may mean it is difficult to detect a change in studies, particularly with a small number of participants and few time points. The provision of ad libitum meals and snacks with known compositions in laboratory settings may seem to be a more accurate method to determine daily energy intake with less potential for underreporting; however, energy intake behaviours could be affected by an artificial environment, social desirability (if eating in the presence of others), and differences in desire for the types of foods provided.

There are a number of factors that could result in individual variability of energy intake compensation in response to chronic exercise, such as biological changes driving appetite [7], psychological factors (restraint and disinhibition), and changing food preferences and food reward (hedonic influence) [8]. Restraint refers to the extent to which individuals restrict their diet in order to control their body weight [59]. Disinhibition refers to the loss of restraint or self-control, which may occur in the presence of forbidden foods, alcohol, and emotional states [60]. Acute exercise has been shown to increase energy intake in unrestrained eaters and decrease energy intake in restrained eaters [61]. Whether exercise intensity influences these psychological factors remains unknown. While we recognize that the physiological stimulus of HIIT and SIT is different and hence may affect appetite and energy intake differently, our metaregression found no differences between modalities of interval training used.

Evidence is now emerging that chronic exercise may improve the sensitivity of appetite hormones to regulate appetite more effectively [62]. Although studies have shown no change in fasting anorexigenic hormones (peptide YY, pancreatic polypeptide, and glucagon-like peptide 1) following 12-week exercise interventions [26, 63, 64], Martins et al. [63] found concentrations of PYY and glucagon-like peptide 1 to be elevated to a greater extent in the exercise group following a meal. Furthermore, Sim et al. [26] have shown that HIIT for 12 weeks reduced energy intake at a test meal, following a high energy preload, thereby eluding to an improvement in appetite regulation. Similarly, fasting acylated ghrelin levels appear to increase [63] or remain stable after long-term exercise [26, 64] but are suppressed to a greater extent in the exercise group following a meal [63]. It has been hypothesized that enhanced suppression of acylated ghrelin and stimulation of anorexigenic hormones postprandial with long-term exercise may improve appetite regulation in individuals who are obese by terminating meals more rapidly and leading to longer between-meal intervals [34]. More research is needed to determine long-term effects of exercise on appetite hormones and potential influence on weight loss. As discussed earlier, other long-term adaptations such as insulin sensitivity may also influence appetite regulation.

Our results showed a significant reduction in bodyweight for HIIT only when compared with a no-exercise control; however, only studies that actually measured energy intake were included in this analysis. Therefore, it is not appropriate to make a conclusion regarding the effect of HIIT/SIT on bodyweight from this subgroup of studies. While no significant changes were observed for bodyweight in MICT versus CON, only four studies were included in the pooled analysis, which limits the ability to draw conclusions. As highlighted earlier, the effect of HIIT compared to MICT on weight loss and fat loss remains unclear. Conflicting results are likely due to a number of factors such as individual variability of outcomes and the heterogeneity of interval training protocols in terms of interval duration and total workload. Furthermore, as participant adherence to the prescribed intensity has been poorly reported, it is unclear whether participants are achieving the intensity targets and hence whether there is a significant difference between groups.

Regardless of whether weight loss targets are achieved, exercise produces an abundance of metabolic and cardiovascular health benefits, including but not limited to improvements in blood pressure, lipid profile, and fasting serum glucose [4]. Furthermore, exercise interventions have been shown to result in significant reductions in visceral adipose tissue even in the absence of changes in body mass and/or waist circumference [26, 65], which may contribute to these health benefits.

A major limitation of this review is the varying level of study quality. Only 44% of studies quantified and reported energy cost of exercise, 44% of studies reported exercise adherence, 56% of studies measured habitual physical activity levels, and 6% of studies reported adherence to training intensities. Without measuring and reporting these outcomes, it is difficult to determine the impact an exercise intervention can have on creating an energy-deficit sufficient to affect energy intake. Therefore, although it is difficult for this review to draw definitive conclusions from the results, it does highlight important methodological considerations.

In conclusion, our results found no compensatory increase in energy intake following a period of HIIT/SIT compared to MICT or no exercise. To date, no studies comparing HIIT with MICT have found significant differences in daily energy intake with time or intervention using self-recorded food diaries or food frequency questionnaires. Studies with alternative dietary assessment with less potential bias may be needed to detect significant differences in energy intake. The multiple-pass 24-hour recall method on multiple days can be associated with less underreporting and reduces potential for participant error and bias. Furthermore, it is imperative that future studies report exercise energy expenditure, exercise adherence, and habitual physical activity as total energy expenditure could significantly influence energy intake response. Additionally, to investigate whether exercise intensity influences energy intake, it is important for studies to report on adherence to the exercise protocols to determine whether participants are achieving the target intensities. Finally, HIIT versus MICT studies investigating individual variation in energy intake and the effect on psychological factors (restraint and disinhibition) and food preferences may provide further insight into long-term changes in eating behaviour with exercise.

## Figures and Tables

**Table 1:** Participant and intervention characteristics of included studies.

Reference	Population	Age (years)	BMI (kg/m^2^)	Duration (weeks)/sessions	HIIT/SIT protocol	MICT protocol	CON	Energy intake method
Ruffino et al. [47]	Male T2DM inactive	55	30.6	8/24 HIIT and 40 MICT	SIT: *n*=16; cycling, supervised ∼2 × 30 sec (all-out sprint) with 4 min active recovery. Total duration = ∼10 min	*n*=16; cycling, supervised continuous (40–55% HRR). Total duration = 30 min	N/A	3-day food diary
Higgins et al. [51]	Female healthy inactive	20	30.3	6/18	SIT: *n*=29; cycling, supervised ∼5–7 × 30 sec (all-out sprint) with 4 min active recovery. Total duration = ∼25–38 min	*n*=23; cycling, supervised continuous (60–70% HRR). Total duration = 20–30 min	N/A	24-hour recalls (3 days, automated)
Sim et al. [26]	Male healthy inactive	30	27.2	12/36	SIT: *n*=12; cycling, supervised 10 × 15 sec (170% VO_2peak_) with 60 sec active recovery (30% VO_2peak_). Total duration = 30–45 min	*n*=12; cycling, supervised continuous (60% VO_2peak_). Total duration = 30–45 min	*n*=10	2-day food diary
Keating et al. [19]	Male/female healthy inactive	43	28.3	12/36	SIT: *n*=13; cycling, supervised 6 × 30–60 sec (120% VO_2peak_) with 2-3 min active recovery. Total duration = 20–24 min	*n*=13; cycling, supervised continuous (50–65% VO_2peak_). Total duration = 36–48 min	*n*=12	3-day food diary
Kong et al. [52]	Female healthy inactive	20	25.7	5/20	HIIT: *n*=10; cycling 60 × 8 sec (90% VO_2max_) with 12 sec active recovery. Total duration = ∼20 min	*n*=8; cycling continuous (65% VO_2peak_). Total duration = 40 min	N/A	3-day food diary
Martins et al. [46]	Male/female healthy inactive	34	33.3	12/36	HIIT: *n*=16; cycling, supervised ∼60 × 8 sec (85–90% VO_2max_) with 12 sec passive rest. Total duration = 20 min	*n*=14; cycling, supervised continuous (70% VO_2peak_). Total duration = 32 min	N/A	3-day food diary
Panissa et al. [24]	Female healthy untrained	28	24.6	6/18	HIIT: *n*=11; unknown mode, supervised 15 × 1 min (90% HR_max_) with 30 sec active recovery (60% HR_max_). Total duration = 22 min	*n*=11; unknown mode, supervised continuous (70% HR_max_). Total duration = 29 min	N/A	3-day food diary
Matsuo et al. [23]	Male metabolic syndrome inactive	48	27.6	8/24	HIIT: *n*=13; cycling, supervised 3 × 3 min (85% VO_2peak_) with 2 min active recovery (50% VO_2peak_). Total duration = 18 min	*n*=13; cycling, supervised continuous (65% VO_2peak_). Total duration = 45 min	N/A	3-day food diary (weighed)
Sasaki et al. [21]	Male healthy inactive	NR	23.9	4/12	HIIT: *n*=12; cycling 10 × 1 min (85% VO_2max_) with 30 sec passive rest. Total duration = 14 min	*n*=12; cycling continuous (45% VO_2peak_). Total duration = 22 min	N/A	Self-administered diet history questionnaire

Earnest et al. [17]	Male metabolic syndrome inactive	48	30.9	6/18	HIIT: *n*=21; treadmill, supervised 8 × 2 min (90–95% VO_2max_) with 2 min active recovery (50% VO_2max_). Total duration = 12 kcal/kg/week	*n*=16; treadmill, supervised continuous (50–70% VO_2max_). Total duration = 12 kcal/kg/week	N/A	Food frequency questionnaire
Eimarieskandari et al. [20]	Female healthy inactive	22	29.6	8/24	HIIT: *n*=7; treadmill 4 × 4 min (85–95% HR_peak_) with 3 min active recovery (50–70% HR_2peak_). Total duration = 33 min	*n*=7; treadmill continuous (50–70% HR_peak_). Total duration = 36–41 min	N/A	7-day diet questionnaire
Wallman et al. [54]	Male/female healthy inactive	42	30.5	8/32	HIIT: *n*=8; cycling, supervised 10 × 60 sec (90% VO_2peak_) with 2 min active recovery (30% VO_2peak_). Total duration = 30 min	*n*=8; cycling, supervised continuous (50–65% VO_2peak_). Total duration = 30 min	*n*=8	7-day food diary
Trapp et al. [18]	Female healthy inactive	20	23.2	15/45	HIIT: *n*=15; cycling 60 × 8 sec (85% VO_2max_) with 12 sec active recovery. Total duration = 20 min	*n*=15; cycling continuous (60% VO_2peak_). Total duration = 40 min	*n*=15	3-day food diary
Heydari et al. [50]	Male healthy inactive	25	29.0	12/36	HIIT: *n*=20; cycling, supervised 60 × 8 sec (85–90% HR_peak_) with 12 sec active recovery. Total duration = 20 min	N/A	*n*=18	3-day food diary
Stensvold et al. [49]	Male/female metabolic syndrome inactive	50	31.8	12/36	HIIT: *n*=11; treadmill, supervised 4 × 4 min (90–95% HR_peak_) with 3 min active recovery (70% HR_peak_). Total duration = 43 min	N/A	*n*=10	Food frequency questionnaire
Walter et al. [53]	Female healthy active	22	23.6	6/18	HIIT: *n*=19; cycling, supervised 5 × 2 min (90–100% HR_max_) with 1 min passive rest. Total duration = 19 min	N/A	*n*=11	24-hour recalls (3 days, manual)

BMI: body mass index; HIIT: high-intensity interval training; SIT: sprint interval training; HR_peak_: peak heart rate; VO_2peak_: peak oxygen consumption; MICT: moderate-intensity continuous training; CON: control.

**Table 2:** Study quality.

Study	1	2	3	4	5	6	7	8	9	10	11	12	13	Total
Earnest et al. [17]	1	1	1	1	0	1	0	1	1	1	1	1	1	11
Eimarieskandari et al. [20]	1	1	0	1	0	1	0	1	1	0	0	0	0	6
Heydari et al. [50]	0	1	0	1	0	0	0	1	1	1	0	0	0	5
Higgins et al. [51]	1	1	0	1	0	1	0	1	1	1	1	1	1	10
Keating et al. [19]	1	1	1	1	1	1	1	1	1	1	1	1	0	12
Kong et al. [52]	1	1	0	1	0	0	0	1	1	0	0	1	1	7
Martins et al. [46]	1	1	0	1	0	0	0	1	1	1	1	1	1	9
Matsuo et al. [23]	1	1	1	1	0	1	0	1	1	1	1	0	1	10
Panissa et al. [24]	1	1	0	0	0	1	0	1	1	1	0	0	0	6
Ruffino et al. [47]	1	1	0	1	0	0	0	1	1	1	1	1	0	8
Sasaki et al. [21]	0	1	0	1	0	0	0	1	1	0	0	1	0	5
Sim et al. [26]	1	1	1	1	0	1	0	1	1	1	1	1	0	10
Stensvold et al. [49]	1	1	1	1	0	1	0	1	1	1	0	0	0	8
Trapp et al. [18]	0	1	0	1	0	0	0	1	1	0	0	0	1	5
Wallman et al. [54]	0	1	0	1	0	1	0	1	1	1	0	1	1	8
Walter et al. [53]	1	1	0	1	0	1	1	1	1	1	0	0	0	8

Modified PEDro criteria: (1) eligibility criteria were specified; (2) participants were randomly allocated to groups; (3) allocation was concealed; (4) groups were similar at baseline; (5) blinding of assessors; (6) measures of at least one key outcome were obtained from more than 85% of participants allocated to groups; (7) intention to treat analysis: data for at least one key outcome were analyzed by “intention to treat”; (8) the results of between-group statistical comparisons are reported for the primary outcome; (9) the study provides the point measures and measures of variability for at least one key outcome; (10) exercise training was supervised; (11) exercise adherence was reported; (12) habitual physical activity was measured; (13) energy expenditure of exercise training was estimated/calculated and reported.

**Table 3:** Metaregression of the pooled effect of comparisons of HIIT/SIT versus MICT on energy intake (kJ) by characteristics of studies.

Comparison	Regression coefficient (95% CI)	*P* value
HIIT/SIT versus MICT (13 studies)		
(i) Duration	−0.2043 (−1.3249 to 0.9163)	0.767
(ii) Sessions	0.0315 (−0.3807 to 0.4437)	0.881
(iii) Sex: male	−0.2832 (−1.2370 to 0.6706)	0.561
(iv) Sex: female	−0.5507 (−1.8521 to 0.7507)	0.407
(v) Age	0.0084 (−0.0468 to 0.0637)	0.765
(vi) BMI	0.0749 (−0.7328 to 0.8826)	0.858
(vii) Metabolic disease	−0.0137 (−0.9263 to 0.8990)	0.977
(viii) Energy intake method	—	0.992
(xi) Study quality	0.3190 (−0.3588 to 0.9968)	0.356
HIIT/SIT versus CON (7 studies)		
(i) Duration	−0.2043 (−1.3249 to 0.9163)	0.721
(ii) Sessions	0.0315 (−0.3807 to 0.4437)	0.881
(iii) Sex: male	1.9613 (−2.2499 to 6.1725)	0.361
(iv) Sex: female	−0.0725 (−5.6154 to 5.4704)	0.980
(v) Age	0.1078 (−0.1153 to 0.3309)	0.344
(vi) BMI	0.0305 (−2.2832 to 2.3443)	0.979
(vii) Metabolic disease	1.1265 (−2.1349 to 4.3879)	0.979
(viii) Energy intake method	—	0.826
(xi) Study quality	1.7705 (−0.3926 to 3.9337)	0.109

BMI: body mass index; HIIT: high-intensity interval training; SIT: sprint interval training; MICT: moderate-intensity continuous training; CON: control; CI: confidence interval.

## Abbreviations

HIIT: High-intensity interval training
SIT: Sprint interval training
MICT: Moderate-intensity continuous training
VO_2peak_: Peak oxygen consumption
HR_peak_: Peak heart rate
PEDro: Physiotherapy Evidence Database
BMI: Body mass index
CI: Confidence interval.
